# Transcatheter Tricuspid Valve Annuloplasty vs Edge-to-Edge Repair: A Propensity-Matched Multicenter Comparative Analysis

**DOI:** 10.1016/j.jacadv.2026.103033

**Published:** 2026-07-21

**Authors:** Jennifer von Stein, Philipp von Stein, Lukas Stolz, Jan Althoff, Caroline Hasse, Sebastian Rosch, Felix Rudolph, Johannes Kirchner, Bjoern Goebel, Benedikt Koell, Wolfgang Rottbauer, Tienush Rassaf, Harald Beucher, Martin Kraus, Mohammad Kassar, Tobias Geisler, Andreas Rück, Joao Ferreira-Martins, Stefan Toggweiler, Paula Sagmeister, Dirk Westermann, Thomas J. Stocker, Ludwig T. Weckbach, Michael Näbauer, Sam Dawkins, Tobias Kister, Marc Vorpahl, Mathias H. Konstandin, Peter Luedike, Mirjam Kessler, Christos Iliadis, Philipp Lauten, Christian Besler, Daniel Kalbacher, Kai Friedrichs, Muhammed Gerçek, Juan F. Granada, Philipp Lurz, Jörg Hausleiter, Mirjam G. Wild, Maria I. Körber, PASTE Investigators, Florian Schindhelm, Tom Cahill, Kornelia Löw, Philipp Schlegel, Norbert Frey, Dominik Felbel, Stephanie Andreß, Amir Abbas Mahabadi, Volker Rudolph, Leonie Ziegler, Cornelia Deutsch, Violetta Hachaturyan, Peter Bramlage, Isabela Kast, Sebastian Ludwig, Roman Pfister, Stephan Baldus, Maria I. Körber

**Affiliations:** a Department of Cardiology, Heart Center, University of Cologne, Cologne, Germany; b Cardiovascular Research Foundation, New York, New York, USA; c Medizinische Klinik und Poliklinik I, LMU Klinikum, LMU München, Munich, Germany; d German Center for Cardiovascular Research (DZHK), Partner Site Munich Heart Alliance, Munich, Germany; e Department of Cardiology, Cardiology I, University Medical Center of the Johannes Gutenberg-University Mainz, Mainz, Germany; f Department of General and Interventional Cardiology/Angiology, Heart and Diabetes Center NRW, Ruhr University Bochum, Germany; g Department of Cardiology, Heart Center, Zentralklinik Bad Berka, Bad Berka, Germany; h Department of Cardiology, University Heart & Vascular Center Hamburg, University Medical Center Hamburg-Eppendorf, Hamburg, Germany; i German Center of Cardiovascular Research (DZHK), Partner Site Hamburg/Kiel/Lübeck; j Department of Cardiology, University Heart Center Ulm, Ulm, Germany; k University Hospital Essen, University Duisburg-Essen, West German Heart- and Vascular Center, Department of Cardiology and Vascular Medicine, Essen, Germany; l Department of Cardiology, Helios Klinikum Siegburg, Germany; m Division of Cardiology, Department of Internal Medicine III, University Hospital Heidelberg, Ruprecht-Karl University Heidelberg, Heidelberg, Germany; n Department of Cardiology, Inselspital Bern, Bern University Hospital, Switzerland; o Medical Clinic III, University Hospital Tübingen, Tübingen, Germany; p Department of Cardiology, Karolinska University Hospital, Stockholm, Sweden; q Oxford Heart Centre, Oxford University Hospitals NHS Foundation Trust, Oxford, United Kingdom; r Heart Center Lucerne, Luzerner Kantonsspital, Lucerne, Switzerland; s Department of Cardiology, Heart Center Leipzig, University of Leipzig, Leipzig, Germany; t University Heart Center Freiburg/Bad Krozingen Bad Krozingen Germany

**Keywords:** annuloplasty, Cardioband, PASTE, tricuspid regurgitation, T-TEER, TTVA

## Abstract

**Background:**

Transcatheter tricuspid valve (TV) interventions are an effective treatment option for patients with severe symptomatic tricuspid regurgitation (TR). TV transcatheter edge-to-edge repair (T-TEER) and transcatheter TV annuloplasty (TTVA) represent distinct repair strategies; however, comparative data remain limited.

**Objectives:**

The objective of the study was to compare procedural and clinical outcomes after T-TEER and TTVA in patients with severe secondary TR.

**Methods:**

Consecutive T-TEER or TTVA-treated patients (2017-2024) were included. A 1:1 propensity-score matching was performed. Endpoints included residual TR ≤ 1+/≤2+, a ≥2-grade reduction, procedural complications, symptomatic improvement, reinterventions, and the composite of all-cause mortality or first heart failure hospitalization within 1 year.

**Results:**

Among 1,122 patients (882 T-TEER; 240 TTVA), 111 well-balanced matched pairs were identified. At discharge, T-TEER achieved higher rates of residual TR ≤ 1+ (50.0% vs 34.2%) and ≤2+ (87.7% vs 67.6%; all *P* < 0.05) compared with TTVA, whereas a ≥2-grade reduction was comparable. Procedural complications were less frequent with T-TEER (any complication: 4.5% vs 27.0% for T-TEER vs TTVA; *P* < 0.001). Symptomatic improvement and reintervention rates within 1 year were similar between groups. Freedom from the composite endpoint of all-cause mortality or first heart failure hospitalization at 1-year was 75.0% (95% CI: 66.8-84.1) after T-TEER and 79.8% (95% CI: 72.4-87.9) after TTVA (*P* = 0.408).

**Conclusions:**

In patients with severe secondary TR and sufficient clinical and anatomical overlap for matched comparison, T-TEER showed a more favorable safety profile and more effective, durable TR reduction, whereas early clinical outcomes at 1 year were comparable after adjustment for baseline differences, highlighting the need for longer-term evaluation.

Moderate or severe tricuspid regurgitation (TR) affects approximately 4% of individuals older than 75 years, with prevalence steadily increasing with age.^1^ In recent years, transcatheter tricuspid valve (TV) interventions have emerged as established treatment options for patients with severe symptomatic TR, and have been associated with improvements in functional capacity and reductions in heart failure hospitalizations (HFHs).^2^^,^^3^

Currently, the most widely adopted repair strategy is TV transcatheter edge-to-edge repair (T-TEER), in which 2 corresponding leaflets are approximated following the Alfieri principle to improve leaflet coaptation. In addition, T-TEER confers an indirect annuloplasty effect, which may favorably influence long-term TR progression.^4^ Transcatheter TV annuloplasty (TTVA) with the Cardioband system (Edwards Lifesciences)—requiring sequential anchor placement along the atrial aspect of the tricuspid annulus (TA) followed by cinching to achieve annular reduction—represented the only alternative catheter-based repair approach to have received CE marking in Europe, although the device was commercially discontinued in 2024 and is no longer available, whereas direct annuloplasty remains a relevant repair concept for future device development.

Despite the different conceptual appeal and the parallel use of both devices over years, direct comparative evidence between T-TEER and TTVA remains scarce. A small retrospective analysis from early experience with both technologies suggested comparable efficacy, albeit with higher bleeding rates in the annuloplasty cohort.^5^ Given the distinct mechanistic approaches and anatomy-driven patient selection, direct comparisons between T-TEER and TTVA remain challenging. However, understanding how these strategies perform in contemporary real-world practice may provide important insights into the relationship between procedural efficacy and clinical outcomes in patients with severe TR. Therefore, we aimed to compare procedural, echocardiographic and clinical outcomes after T-TEER and TTVA in patients with symptomatic severe secondary TR, including a propensity-score matched (PSM) analysis to account for baseline differences.

## Methods

### Study design and population

This study included consecutive patients with symptomatic severe TR from 2 independent European cohorts of transcatheter TV repair: the PASTE registry (PASCAL for Tricuspid Regurgitation) and a multicenter TTVA cohort. Patients with isolated primary TR were excluded.

The PASTE registry is an investigator-initiated, multicenter, retrospective, and prospective observational study conducted across 16 European heart valve centers, including all-comers undergoing T-TEER with the PASCAL repair system (Edwards Lifesciences) between 2019 and 2023.^6^ The study was approved by the local ethics committees and registered at ClinicalTrials.gov NCT05328284.

The TTVA cohort comprised consecutive patients who were treated with the Cardioband at 3 high-volume centers in Germany (Heart Center at the University Hospital of Cologne, Heart and Diabetes Center North Rhine-Westphalia in Bad Oeynhausen, and University Heart and Vascular Center in Hamburg) between 2017 and 2024.

Treatment allocation was driven by anatomical suitability and local heart team decision-making, with TTVA preferentially performed in patients with anatomical features limiting feasibility of T-TEER, as previously described.^7^ Data were collected retrospectively after Institutional Review Board approval.

### Echocardiographic assessment

Echocardiographic evaluation was performed according to current guideline recommendations for the assessment of TR and right heart function.^8^^,^^9^ For the PASTE registry, analyses were centralized, whereas in the TTVA cohort they were performed locally by dedicated echocardiography specialists using the same guideline-based grading framework across participating centers. TR severity was graded following the five-grade quantification scheme, including the grades none (0+), mild (1+), moderate (2+), severe (3+), massive (4+), and torrential (5+).

Right-sided chamber size and function were assessed by conventional parameters including right ventricular (RV) base diameter (RVBD), right atrial (RA) area, TA diameter, and tricuspid annular plane systolic excursion (TAPSE). TV morphology was graded according to the recently proposed nomenclature, with complex morphology defined as >3 leaflets.^10^

An atrial phenotype was defined by TAPSE >17 mm, left ventricular ejection fraction (LVEF) ≥50%, and tenting height <10 mm, whereas a ventricular phenotype was defined as not meeting 1 of these criteria.

### Study endpoints and outcomes

The primary endpoint was the proportion of patients achieving residual TR ≤ 1+ or ≤2+ at discharge and follow-up, as well as a ≥2-grade TR reduction from baseline to discharge.

Secondary endpoints included intraprocedural success, defined according to the Tricuspid Valve Academic Research Consortium criteria;^11^ procedural complications including single-leaflet device attachment or TTVA-anchor detachment, access-site related vascular injury, major bleeding, acute kidney injury (AKI), coronary artery injury requiring percutaneous coronary intervention (PCI), and stroke; changes in echocardiographic parameters from baseline to follow-up, including LVEF, TAPSE, RVBD, and RA area; durability of TR reduction, defined as loss of efficacy (LOE) among patients with residual TR ≤2+ at discharge and TR ≥3+ at follow-up; symptomatic improvement assessed by NYHA functional class from baseline to follow-up; TV reintervention within 1 year; and the composite of all-cause mortality or first HFH within 1 year. Endpoints were assessed in both the overall cohort and the PSM cohort.

### Statistical analysis

Continuous variables are presented as mean ± SD or median (Q1–Q3), and categorical variables as frequencies (n [%]). Between-group comparisons were performed using the chi-square test, Student’s t-test, or Mann-Whitney U test, as appropriate. Paired comparisons were assessed with the Wilcoxon signed rank test or McNemar test. Differences in echocardiographic parameters at follow-up were analyzed using analysis of covariance with baseline values as covariates. Effect modification by treatment strategy was assessed using baseline-adjusted linear models with interaction terms.

To reduce confounding related to baseline differences, a 1:1 nearest neighbor PSM was performed without replacement using a caliper of 0.2 of the SD of the logit of the propensity score. Covariates were selected based on clinical relevance and completeness (≤25% missingness), and included: age, sex, body mass index, NYHA functional class, history of myocardial infarction, aspartate aminotransferase, N-terminal pro-B-type natriuretic peptide, TR etiology, LVEF, left ventricular end-diastolic diameter, baseline TR grade, baseline mitral regurgitation grade, TAPSE, RVBD, TA diameter, RA area, inferior vena cava, pulmonary artery systolic pressure, and TV morphology. Balance was assessed using standardized mean differences, with <0.1 indicating excellent balance.

To further assess robustness, sensitivity analyses were performed using inverse probability of treatment weighting with stabilized weights and a stricter propensity score matching caliper of 0.1, applying the same covariates and reassessing the composite endpoint.

Time-to-event analysis for the composite endpoint of all-cause mortality and first HFH was performed using Kaplan-Meier estimates. A 2-sided *P* value < 0.05 indicated statistical significance. Analyses were conducted using R (version 4.4.0; R Foundation for Statistical Computing).

## Results

### Baseline characteristics

Among 1,122 included patients, 882 underwent T-TEER with the PASCAL system and 240 TTVA. Patients treated with TTVA were younger and more frequently females, with a higher prevalence of prior HFH. In contrast, patients undergoing T-TEER had higher N-terminal pro-B-type natriuretic peptide levels, greater diuretic requirements, and slightly higher European System for Cardiac Operative Risk Evaluation II values. Baseline characteristics are summarized in Table 1.

**Table 1:** Baseline Unmatched Characteristics of T-TEER and TTVA

Characteristic	T-TEER (n = 882)	TTVA (n = 240)	*P* Value
Age, y	81 (76-84)	79 (75-82)	**0.001**
Female, %	458/882 (51.9)	183/240 (76.2)	**<0.001**
BMI, kg/m^2^	25.1 (22.4-28.4)	26.0 (22.8-30.0)	0.051
TRI-SCORE	4 (3-5) (n = 882)	4 (3-6) (n = 240)	0.088
EuroScore II, %	4.8 (3.0-8.6) (n = 848)	4.1 (2.9-7.3) (n = 240)	**0.045**
NYHA functional class			0.115
I	6/882 (0.7)	0/240 (0)	
II	128/882 (14.5)	30/240 (12.5)	
III	652/882 (73.9)	193/240 (80.4)	
IV	96/882 (10.9)	17/240 (7.1)	
Prior HFH	490/872 (56.2)	146/220 (66.4)	**0.008**
Comorbidities			
Atrial fibrillation/flutter	805/882 (91.3)	218/240 (90.8)	0.934
Coronary artery disease	340/881 (38.6)	96/240 (40)	0.748
RV lead	250/878 (28.5)	53/240 (22.1)	0.059
Prior myocardial infarction	84/879 (9.6)	10/197 (5.1)	0.061
History of cardiac surgery	194/878 (22.1)	56/240 (23.3)	0.749
COPD	136/882 (15.4)	43/240 (17.9)	0.402
Laboratory data			
NT-proBNP, pg/mL	2,459.0 (1,380.0-4,815.0) (n = 821)	2059.5 (1,328.0-3,935.3) (n = 226)	**0.011**
AST, U/l	26.4 (20.4-33.6) (n = 846)	29.0 (25.0-36.0) (n = 229)	**<0.001**
eGFR, mL/min/1.73 m^2^	42 (32-56)	43 (33-58)	0.257
eGFR <60 mL/min	706/882 (80)	184/240 (76.7)	0.291
Medication			
Loop diuretic agents	821/882 (93.1)	191/208 (91.8)	0.629
Furosemide-equivalent, mg	60 (40-120) (n = 828)	40 (20-100) (n = 208)	**0.001**
Hemodynamic data			
PASPinv, mm Hg	42 (35-52) (n = 594)	41 (35-48) (n = 177)	**0.034**

Loop diuretic doses were expressed as furosemide-equivalents (mg) using standard conversion factors (torsemide × 4). Values are n (%), median [Q1-Q3], or mean ± SD. **Bold** values indicate statistical significance.

AST = aspartate aminotransferase; BMI = body mass index; COPD = chronic obstructive pulmonary disease; eGFR = estimated glomerular filtration rate; EuroSCORE II = European System for Cardiac Operative Risk Evaluation II; HFH = heart failure hospitalization; NT-proBNP = N-terminal pro–B-type natriuretic peptide; PASPinv = invasively measured pumonary artery systolic pressure; RV = right ventricular; T-TEER = tricuspid valve transcatheter edge-to-edge repair; TTVA = transcatheter tricuspid valve annuloplasty.

### Baseline echocardiographic assessment

Both groups demonstrated right heart enlargement, with TTVA patients showing slightly smaller RV dimensions, but larger TA diameters. Although the overall prevalence of massive or torrential TR was similar, torrential TR occurred more frequently in the TTVA group (*P* < 0.001). Leaflet tethering and coaptation gap width were comparable between groups, whereas complex TV morphology (types III–IV) was more prevalent among TTVA patients (56% vs 44%; *P* = 0.016). Baseline echocardiographic characteristics are summarized in Table 2.

**Table 2:** Baseline Unmatched Echocardiographic Assessment of T-TEER and TTVA

Parameter	T-TEER (n = 882)	TTVA (n = 240)	*P* Value
RAA, cm^2^	36.0 (29.0-44.0) (n = 826)	34.9 (28.7-42.1) (n = 234)	0.168
RV base diameter, mm	50.0 (44.0-58.0) (n = 835)	47.3 (43.0-53.0) (n = 234)	**<0.001**
TAPSE, mm	17 (15-20) (n = 820)	16 (14-19) (n = 235)	**0.009**
Estimated PASP, mm Hg	41 (33-52) (n = 776)	35 (27-44) (n = 233)	**<0.001**
Inferior vena cava, mm	24 (20-29) (n = 775)	26 (21-30) (n = 205)	**0.041**
Tricuspid valve			
Coaptation gap, mm	5.3 (4.0-8.0) (n = 823)	6.0 (3.0-10.0) (n = 190)	0.286
Tenting height, mm	6.2 (4.5-8.9) (n = 798)	6.5 (4.4-9.0) (n = 201)	0.415
Annular dimension SL, mm	41 (35-47) (n = 848)	43 (40-47.2) (n = 193)	**<0.001**
Morphology			**0.016**
I	376/706 (53.3)	72/179 (40.2)	
II	17/706 (2.4)	6/179 (3.4)	
IIIa	30/706 (4.2)	9/179 (5)	
IIIb	216/706 (30.6)	63/179 (35.2)	
IIIc	31/706 (4.4)	17/179 (9.5)	
IV	36/706 (5.1)	12/179 (6.7)	
Tricuspid regurgitation			
Grade			**<0.001**
0	0/0 (0.0)	0/0 (0.0)	
1+	0/0 (0.0)	0/0 (0.0)	
2+	0/0 (0.0)	0/0 (0.0)	
3+	363/882 (41.2)	97/240 (40.4)	
4+	326/882 (37)	62/240 (25.8)	
5+	193/882 (21.9)	81/240 (33.8)	
Vena contracta width, mm	13.0 (10.0-16.5) (n = 876)	13.0 (10.0-17.0) (n = 231)	0.640
EROA, cm^2^	0.61 (0.45-0.82) (n = 782)	0.67 (0.5-0.91) (n = 231)	**0.033**
Regurgitant volume, mL	49 (37-63) (n = 768)	49 (35-66) (n = 217)	0.919
Etiology			**0.010**
Primary	0/0 (0.0)	0/0 (0.0)	
Secondary	777/882 (88.1)	226/240 (94.2)	
Mixed	105/882 (11.9)	14/240 (5.8)	
Secondary TR etiology			0.130
A-STR	203/659 (30.8)	46/186 (24.7)	
V-STR	456/659 (69.2)	140/186 (75.3)	
Left Ventricle			
LVEF, %	55 (45-60) (n = 854)	55 (49-59) (n = 238)	0.735
LVEDD, mm	48 (43-53) (n = 786)	44 (40-49) (n = 202)	**<0.001**
Mitral regurgitation grade			
0	211/858 (24.6)	21/205 (10.2)	**<0.001**
1+	484/858 (56.4)	125/205 (61)	
2+	130/858 (15.2)	53/205 (25.9)	
3+	32/858 (3.7)	6/205 (2.9)	
4+	1/858 (0.1)	0/205 (0)	

Values are n (%), median (Q1-Q3), or mean ± SD. **Bold** values indicate statistical significance.

A-STR = atrial secondary tricuspid regurgitation; EROA = effective regurgitant orifice area; LVEF = left ventricular ejection fraction; LVEDD = left ventricular end diastolic diameter; PASP = pulmonary artery systolic pressure; RAA = right atrial area; SL = septolateral; TAPSE = tricuspid annular plane systolic excursion; TR = tricuspid regurgitation; V-STR = ventricular secondary tricuspid regurgitation; other abbreviations as in Table 1.

### Procedural and safety outcomes

Procedural duration was substantially longer with TTVA compared with T-TEER (197 vs 105 minutes; *P* < 0.001), whereas intraprocedural success was achieved more frequently with T-TEER (84.3% vs 65.0%; *P* < 0.001). T-TEER resulted in higher rates of residual TR ≤ 1+ and ≤2+ at discharge (all *P* < 0.001). A ≥2-grade TR reduction was achieved in 76.8% and 70.6% of patients after T-TEER and TTVA, respectively (*P* = 0.063).

Major safety events, including device-related complications such as single-leaflet device attachment and partial Cardioband detachment, were infrequent and occurred less often after T-TEER. Rates of major bleeding, AKI, stroke, and coronary injury were lower with T-TEER, resulting in a lower overall complication rate compared with TTVA (Table 3).

**Table 3:** Procedural and Safety Outcomes in Unmatched T-TEER and TTVA

Variable	T-TEER (n = 882)	TTVA (n = 240)	*P* Value
Procedure time, min	105 (76-147) (n = 829)	197 (166-244) (n = 227)	**<0.001**
Intraprocedural success	740/878 (84.3)	156/240 (65.0)	**<0.001**
TR grade			**<0.001**
0	63/826 (7.6)	22/238 (9.2)	
1+	343/826 (41.5)	65/238 (27.3)	
2+	282/826 (34.1)	76/238 (31.9)	
3+	119/826 (14.4)	54/238 (22.7)	
4+	19/826 (2.3)	15/238 (6.3)	
5+	0/826 (0)	6/238 (2.5)	
Number of devices	2 (1-2)	b	
Device type (PASCAL)		b	
PASCAL P10	140/882 (15.9)		
PASCAL Ace	742/882 (84.1)		
Device position		b	
AS only	420/804 (52.2)		
PS only	61/804 (7.6)		
AS and PS combined	481/804 (59.8)		
Concomitant M-TEER	51/882 (5.8)	0/206 (0)	**0.001**
Number of anchors	b	17 (16-17) n = 215	
Device type (Cardioband)	b		
C		3/228 (1.2)	
D		21/228 (8.8)	
E		56/228 (23.3)	
F		148/228 (61.7)	
Degree of cinching	b	5.5 (5.5-5.5) n = 224	
Patients with any safety event	43/882 (4.9)	52/240 (21.7)	**<0.001**
Total number of safety events	45	67	
SLDA^a^	34/882 (3.9)	b	
Anchor-detachment^c^	b	3/240 (1.2)	
Access-site related venous or arterial injury (TVARC type ≤3 bleeding)	4/882 (0.5)	4/240 (1.7)	0.122
Bleeding (TVARC type >3)	5/882 (0.6)	12/240 (5.0)	**<0.001**
Stroke/TIA	0/882 (0)	3/240 (1.2)	**0.009**
Acute kidney injury	2/880 (0.2)	14/240 (5.8)	**<0.001**
RCA perforation	b	19/240 (7.9)	
RCA PCI	b	11/240 (4.6)	
Acute myocardial infarction	0/882 (0)	1/240 (0.4)	0.485

Values are n (%). **Bold** values indicate statistical significance.

AS = anteroseptal; M-TEER = mitral valve transcatheter edge-to-edge repair; PCI = percutaneous coronary intervention; PS = posteroseptal; RCA = right coronary artery; SLDA = single-leaflet device attachment; TIA = transient ischemic attack; TVARC = Tricuspid Valve Academic Research Consortium; other abbreviations as in Tables 1 and 2.

a Variable observed only in 1 treatment cohort; between-group comparison not applicable.

b SLDA was identified intraprocedurally.

c Anchor-detachment was identified at discharge.

### Propensity-score matched cohort

To account for baseline differences, 1:1 propensity score matching yielded 111 well-balanced patient pairs (Supplemental Figure 1, Supplemental Tables 1 and 2).

### Procedural and safety outcomes

Intraprocedural success remained more frequent with T-TEER. At discharge, residual TR ≤ 1+ and ≤2+ were achieved more often after T-TEER than after TTVA (Central Illustration). A ≥2-grade TR reduction was observed in 79.2% (84/106) and 66.7% (74/111) of patients after T-TEER and TTVA, respectively (*P* = 0.054).

**Central Illustration:**
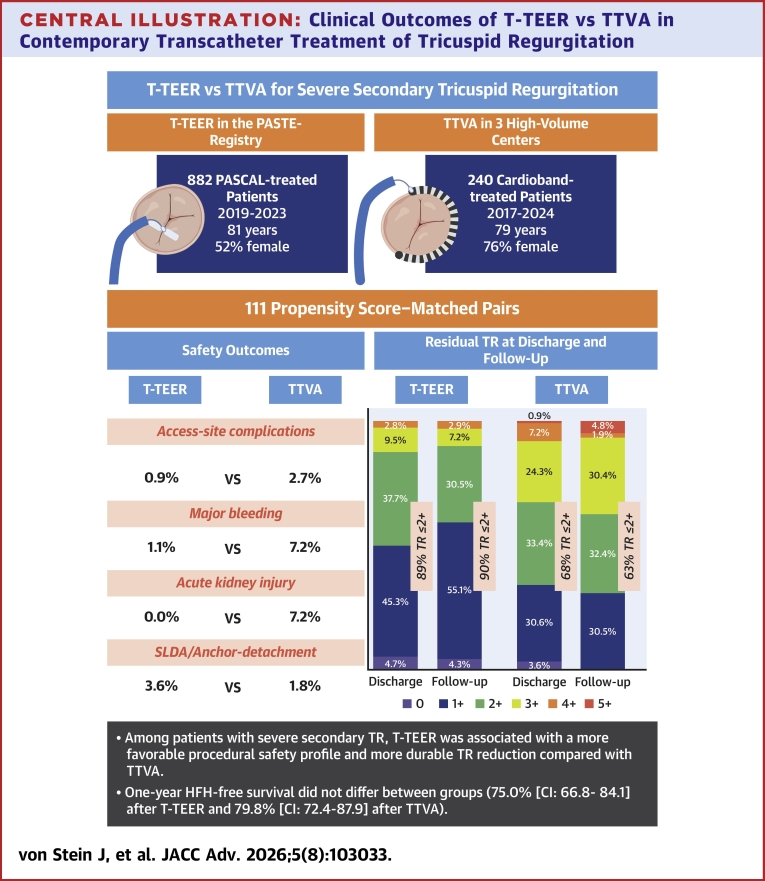
**Clinical Outcomes of T-TEER vs TTVA in Contemporary Transcatheter Treatment of Tricuspid Regurgitation** In 111 propensity score–matched patient pairs with severe secondary TR, T-TEER demonstrated a more favorable procedural safety profile and achieved higher rates of residual TR ≤1+ and ≤2+ at discharge and follow-up compared with TTVA. At discharge, residual TR ≤1+ was observed in 53/106 patients (50.0%) after T-TEER and 38/111 (34.2%) after TTVA, and residual TR ≤2+ in 93/106 (87.7%) and 75/111 (67.6%), respectively. At follow-up (median 365 [203-445] days after T-TEER and 373 [65-588] days after TTVA), residual TR ≤1+ was present in 41/69 (59.4%) and 32/105 (30.5%), and residual TR ≤2+ in 62/69 (89.9%) and 66/105 (62.9%), respectively. Despite differences in TR reduction, freedom from the composite of all-cause mortality or first HFH was comparable between strategies. HFH = heart failure hospitalization; SLDA = single-leaflet device attachment; T-TEER = tricuspid valve transcatheter edge-to-edge repair; TR = tricuspid regurgitation; TTVA = transcatheter tricuspid valve annuloplasty.

Major safety events occurred more frequently after TTVA, with higher rates of major bleeding and AKI. Coronary artery complications, including right coronary artery (RCA) perforation and subsequent PCI, were observed exclusively in the TTVA group (Supplemental Table 3).

### Follow-up and clinical outcomes

At a median follow-up of 365 days (203–445) after T-TEER and 373 days (65–588) after TTVA, residual TR ≤1+ was observed in 59.4% and 30.5%, and residual TR ≤2+ in 89.9% and 62.9% (both *P* < 0.001) (Central Illustration). Among patients with residual TR ≤2+ at discharge, LOE occurred in 22.9% after TTVA and 4.9% after T-TEER (*P* = 0.005). Baseline characteristics of matched T-TEER patients with vs without follow-up echocardiography are shown in Supplemental Table 4.

**Figure 1:**
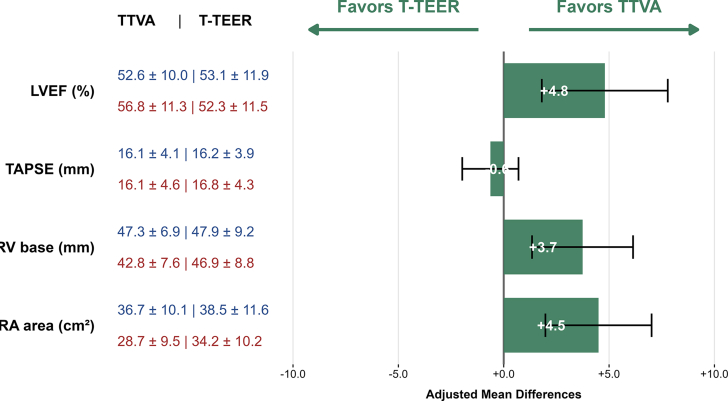
**Baseline-Adjusted Changes in Echocardiographic Parameters After T-TEER and TTVA** Baseline-adjusted echocardiographic changes from baseline (blue) to follow-up (red) comparing TTVA and T-TEER in the matched cohort. TTVA-treated patients had a greater increase in LVEF (+4.8%; 95% CI: 1.8%-7.8%; *P* = 0.002), a greater reduction in RVBD (−3.7 mm; 95% CI: −6.1 to −1.3; *P* = 0.002) and RA area (−4.5 cm^2^; 95% CI: −7.0 to −2.0; *P* < 0.001), whereas changes in TAPSE (−0.6 mm; 95% CI: −2.1 to 0.7; *P* = 0.350) did not differ between strategies. Green bars represent adjusted mean difference with 95% CIs. For RVBD and RA area, values are inverted (positive values represent greater reduction). LVEF = left ventricular ejection fraction; RA = right atrial; RV = right ventricle; TAPSE = tricuspid annular plane systolic excursion; T-TEER = tricuspid valve transcatheter edge-to-edge repair; TTVA = transcatheter tricuspid valve annuloplasty.

Baseline-adjusted echocardiographic analysis showed higher LVEF, smaller RVBD and RA area at follow-up after TTVA, whereas TAPSE did not differ between groups (Figure 1). Although TR reduction was greater with T-TEER (median 2 grades [2-3] vs 2 grades [1-2], *P* = 0.003), treatment strategy did not modify the association between TR reduction and echocardiographic changes (interaction *P* values: LVEF 0.611, TAPSE 0.391, RV base 0.265, RA area 0.395).

**Figure 2:**
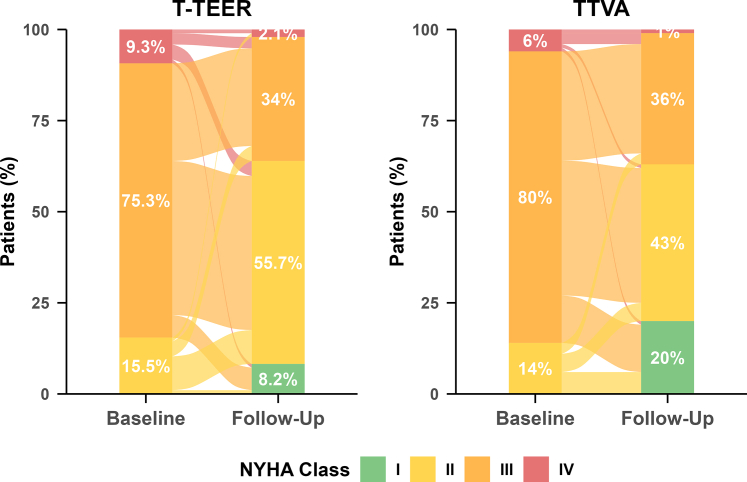
**Changes in NYHA Functional Class From Baseline to Follow-Up After T-TEER and TTVA in the Matched Cohort** Distribution of NYHA functional class at baseline and follow-up for patients undergoing T-TEER (left) and TTVA (right). NYHA functional class ≤II increased from 15.5% to 63.9% after T-TEER and from 14% to 63% after TTVA. No significant difference in NYHA functional class improvement was observed between groups (chi-square *P* = 0.889). Abbreviations as in Figure 1.

Functional status improved significantly in both groups (*P* < 0.001 for both), with NYHA functional class improvement in 57.7% after T-TEER and 61.0% after TTVA (*P* = 0.748) (Figure 2).

Reinterventions within 1 year were rare and did not differ between groups (2 [1.8%] vs 3 [2.7%] events; *P* > 0.999). Individual reintervention trajectories are provided in Supplemental Table 5.

**Figure 3:**
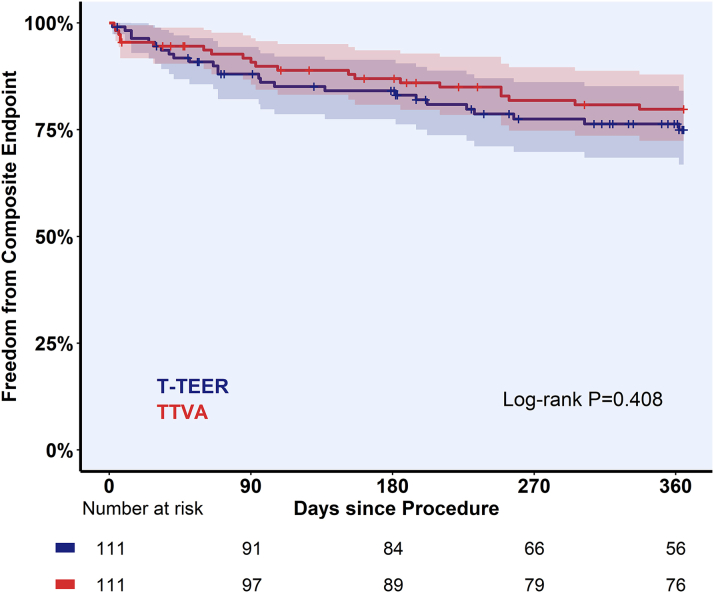
**Freedom From the Composite Endpoint After T-TEER and TTVA in the Matched Cohort** Kaplan-Meier curves depicting freedom from the composite endpoint of all-cause mortality or first heart-failure hospitalization following T-TEER (blue) and TTVA (red). At 1 year, freedom from the composite endpoint was 75.0% (95% CI: 66.8%-84.1%) after T-TEER, compared with 79.8% (95% CI: 72.4%-87.9%) after TTVA. Shaded areas represent 95% CIs. Abbreviations as in Figure 1.

Freedom from the composite of all-cause mortality or first HFH at 1 year was 75.0% and 79.8%, respectively, without significant difference (log-rank *P* = 0.408) (Figure 3). Findings were consistent in sensitivity analyses using inverse probability of treatment weighting and a stricter matching caliper of 0.1 (Supplemental Figures 2–4).

## Discussion

In this large-scale comparative analysis of 2 contemporary transcatheter repair strategies for severe secondary TR, several important observations emerge: 1) T-TEER was associated with a more favorable procedural safety profile, whereas TTVA involved longer procedures and higher rates of complications, including major bleeding, AKI, stroke, and RCA–related events; 2) T-TEER achieved more effective and durable TR reduction, as reflected by higher rates of residual TR ≤ 1+ and ≤2+; 3) despite these differences, clinical outcomes—including symptomatic improvement, reintervention, and HFH-free survival—were comparable between strategies; and 4) longitudinal echocardiographic assessment showed higher LVEF and smaller RV and RA dimensions after TTVA, whereas TAPSE was comparable between strategies.

### Baseline anatomy and implications for treatment

In contemporary practice, T-TEER and TTVA are applied in distinct patient populations, largely determined by anatomical suitability, procedural complexity, and safety considerations, with T-TEER more rapidly adopted. Key factors guiding treatment selection include coaptation gap width, leaflet anatomy, chordal complexity, and jet origin.

Consistent with this paradigm, patients undergoing TTVA more frequently exhibited complex TV morphology, larger annular diameters, and a higher prevalence of torrential TR before matching, despite comparable right heart size, function, coaptation gap width, and tenting height. These findings are consistent with real-world anatomical selection rather than more advanced right-sided disease.

After PSM, clinical and echocardiographic characteristics, including key anatomical parameters, were well balanced, allowing a more direct comparison within the subset of patients with sufficient clinical and anatomical overlap between both treatment strategies. In this matched cohort, T-TEER achieved higher rates of residual TR ≤ 1+ and ≤2+ at discharge and follow-up, consistent with contemporary registries.^12^^,^^13^ Corresponding results after TTVA paralleled prior prospective annuloplasty data,^14^ underscoring fundamental differences in achievable TR reduction between strategies.

These findings underscore the central role of anatomical selection in contemporary practice. Ultimately, treatment pathways continue to evolve, with patients anatomically unsuitable for T-TEER now increasingly referred for transcatheter valve replacement rather than annuloplasty. Accordingly, the present analysis should be interpreted as a comparison of treatment strategies applied to anatomically selected patient populations rather than a direct comparison of interchangeable interventions.

### Procedural complexity and safety

Procedural complexity and safety differed fundamentally between strategies. In the unmatched cohort, TTVA was associated with higher rates of major bleeding, AKI, neurological events, and RCA-related events compared with T-TEER. These differences likely reflect both real-world patient selection and inherent procedural characteristics, as annuloplasty requires prolonged procedural duration (197 vs 105 minutes), extensive annular manipulation, and routine coronary angiography to monitor the RCA during the procedure. The placement of multiple annular anchors carries an intrinsic risk of RCA injury, which may occur because of the close anatomical relationship between the RCA and the TA and may be exacerbated during cinching. In the present study, RCA injury occurred in 19 TTVA-treated patients and was identified intraprocedurally; bailout PCI was performed in 11 patients with clinically relevant or flow-limiting RCA compromise.

PSM harmonized baseline characteristics but did not mitigate procedural risks. In the matched cohort, AKI and major bleeding remained more frequent after annuloplasty, indicating that these safety concerns are intrinsic to the procedure, consistent with prior reports.^15^

### Residual tricuspid regurgitation and clinical outcomes

T-TEER was associated with more durable TR reduction, reflected by higher rates of residual TR ≤2+ at follow-up and a markedly lower incidence of LOE compared with TTVA. This difference may relate to the leaflet-based mechanism of T-TEER, which directly addresses malcoaptation at the dominant regurgitant jet, whereas annuloplasty primarily modifies annular geometry. In addition, postprocedural TR quantification may differ between strategies, as T-TEER devices can render TR grading more challenging compared with annuloplasty, where leaflet anatomy and regurgitant jets often remain more clearly delineated.

Despite these differences in the extent and durability of TR reduction, 1-year clinical outcomes were comparable. This apparent dissociation between procedural efficacy and early clinical outcomes represents a central finding of the present analysis. Several factors may have contributed to this observation. First, the overall magnitude of TR reduction was broadly comparable between strategies, despite differences in residual TR grades, which may partly explain the similar symptomatic improvement.^16^ Second, the advanced disease stage of the present cohort may have attenuated the early clinical translation of more complete residual TR reduction. Compared with patients enrolled in randomized T-TEER trials such as TRILUMINATE, both cohorts exhibited a higher burden of advanced heart failure, with a greater prevalence of NYHA functional class III/IV symptoms (86% and 87% vs 56%), more frequent prior HFH (64% and 61% vs 25%), and more pronounced renal dysfunction (estimated glomerular filtration rate 43 and 44 vs 56 mL/min/1.73 m^2^). The Tri.Fr echocardiographic substudy further supports this concept by showing that clinical improvement at 1 year was associated with a more favorable right-sided remodeling profile, particularly lower RA volume, underscoring the relevance of disease stage beyond residual TR grade alone.^12^ Third, a 1-year follow-up period may be insufficient to capture delayed effects of more durable TR reduction on heart failure events or survival. These observations are consistent with contemporary randomized T-TEER data, including TRILUMINATE and Tri.Fr, which support symptomatic and health-status improvement after TR reduction, whereas longer-term data are needed to define the impact of residual TR thresholds on HFH and mortality. Nevertheless, residual confounding cannot be excluded, and the matched sample size may have limited power to detect modest differences in clinical outcomes.

Longitudinal echocardiography revealed selected differences between strategies, with higher LVEF and smaller basal RV and RA dimensions after TTVA, whereas TAPSE was similar. These findings should be interpreted cautiously, because the available registry parameters did not allow comprehensive assessment of RV remodeling, including three-dimensional RV volumes. Moreover**,** circumferential annular cinching may mechanically reduce RVBD, limiting its value as a marker of reverse remodeling. Interaction analyses demonstrated no differential association between TR reduction and echocardiographic changes across treatment strategies, supporting a broadly similar remodeling response per grade of TR reduction. Accordingly, the observed differences in selected echocardiographic parameters may reflect a combination of treatment mechanism, measurement effects, and unmeasured factors rather than a clearly superior remodeling effect of either strategy.

### Transcatheter annuloplasty in context of surgical principles

TTVA is conceptually rooted in surgical repair, where ring annuloplasty has long been favored over suture-based techniques.^17^ However, the favorable surgical experience cannot be directly extrapolated to the transcatheter setting. Surgical repair is typically performed in younger, lower-risk patients under controlled operative conditions, whereas TTVA targets an older, comorbid population in whom procedural duration and invasiveness may critically influence outcomes.

Although T-TEER has traditionally been conceptualized as a predominantly leaflet-based, jet-oriented therapy, emerging evidence suggest an additional indirect annuloplasty effect, particularly when combined anteroseptal and posteroseptal device configurations—referred to as the *clover strategy*^18^—are employed, with potential clinical implications.^4^ Compared with T-TEER, TTVA generally achieves larger TA reductions of approximately 15% to 20%,^14^^,^^19^ whereas T-TEER is associated with more modest reductions of ∼6–12%.^4^^,^^20^ However, the present analysis cannot definitively compare annular remodeling between strategies, as follow-up annular measurements were not systematically available.

### Strengths and limitations

This study represents the largest comparative analysis to date of T-TEER and TTVA for severe secondary TR, integrating 2 contemporary European cohorts with detailed clinical and echocardiographic characterization. The use of PSM incorporating both clinical and anatomical variables enabled a balanced comparison and mitigated confounding related to real-world treatment selection. Consistency of the primary endpoint across sensitivity analyses further supports the robustness of the findings.

Several limitations warrant consideration. The retrospective observational design precludes causal inference, and residual confounding cannot be excluded. Echocardiographic assessment was centralized for the T-TEER cohort but locally performed for TTVA, potentially introducing variability in residual TR grading despite the use of a guideline-based, standardized multiparametric approach across participating centers. In addition, follow-up echocardiography was less frequently available in the T-TEER cohort, which may have introduced selection bias in the assessment of TR durability. The TTVA experience reflects a device no longer commercially available, which may limit direct generalizability, although the mechanistic insights remain relevant for future technologies. Finally, T-TEER was performed exclusively with the PASCAL system, and results may not extend to other edge-to-edge devices.

## Conclusions

In this matched comparison of transcatheter repair strategies for severe secondary TR among patients with sufficient clinical and anatomical overlap, T-TEER demonstrated a more favorable procedural safety profile and achieved more effective and durable TR reduction than TTVA. Despite these procedural differences, adjusted 1-year clinical outcomes were comparable. In an elderly and comorbid transcatheter TV intervention population, these findings highlight the importance of procedural safety and durable TR reduction, while underscoring the complexity of translating procedural success into early clinical benefit and the need for longer-term evaluation.

Perspectives **COMPETENCY IN PATIENT CARE AND PROCEDURAL SKILLS:** In patients with severe secondary TR, contemporary transcatheter treatment selection should be individualized according to valve anatomy, procedural risk, and the likelihood of achieving durable TR reduction. Although dedicated transcatheter annuloplasty is currently not commercially available, the present findings provide clinically relevant insight into the trade-off between repair efficacy, procedural complexity, and safety, supporting careful differentiation between patients suitable for T-TEER and those who may require transcatheter valve replacement.

**TRANSLATIONAL OUTLOOK:** Further studies with longer-term follow-up are needed to determine whether more complete and durable TR reduction translates into delayed reductions in HFH and mortality. Future comparative studies should integrate standardized echocardiographic adjudication, advanced right heart imaging, and contemporary transcatheter replacement strategies to better define patient- and anatomy-specific treatment pathways for severe secondary TR.

## Funding support and author disclosures

Among the full cohort of patients in the registry, data collection for the Hamburg patients was supported by a grant from the 10.13039/501100005971German Heart Foundation. Dr F. Rudolph received funding from Bielefeld University (clinician scientist entry fellowship). Dr J. von Stein received Lecture honoraria from Edwards Lifesciences. Dr Körber received travel support from JenaValve and lecture fees from Edwards Lifesciences, Abbott, and Siemens Healthineers. Dr Wild received speaker fees from Abbott Vascular and Edwards Lifesciences as well as honoraria for consultancy from IPPMed. Dr Stolz received speaker honoraria from Edwards Lifesciences. Dr Gerçek received consulting fees from Edwards and funding from the Ruhr University Bochum (Advanced Clinician Scientist). Dr Luedike received speaker fees from Edwards Lifesciences. Dr Rassaf received speaker fees from AstraZeneca, Daiichi-Sankyo, Bayer, Novartis, and Abiomed outside the submitted work. Dr Kalbacher received personal fees from Abbott, Edwards Lifesciences, Medtronic Inc., and Pi-Cardia Ltd. Dr Lurz has received grants from Abbott Vascular, Edwards Lifesciences, and ReCor Medical. Dr Toggweiler received honoraria from Medtronic, Boston Scientific, Biosensors, Edwards Lifesciences, Microport, Meril, P + F, Hi-D Imaging, Abbott Vascular, Medira, Shockwave, Teleflex, atHeart Medical, Cardiac Dimensions, Polares Medical, Amarin, Sanofi, AstraZeneca, ReCor Medical, Daiichi Sankyo, Bayer, and Armira; has received institutional research grants from Medtronic, Edwards Lifesciences, Abbott Vascular, Boston Scientific, Fumedica, Novartis, Boehringer Ingelheim, and Polares Medical; and holds equity in Hi-D Imaging. Dr Stocker received speaker honoraria from Edwards Lifesciences; and served as consultant for Occlutech International. Dr Hausleiter received research support and speaker honoraria from Edwards Lifesciences. All other authors have reported that they have no relationships relevant to the contents of this paper to disclose.

## Supplementary materials

Supplemental Material

PASTE Investigator List
