# Supplementary material for: Transcatheter Tricuspid Valve Annuloplasty vs Edge-to-Edge Repair: A Propensity-Matched Multicenter Comparative Analysis
Source: JACC Adv. 2026 Jul 21;5(8):103033. doi: 10.1016/j.jacadv.2026.103033 (PMC13396854; doi:10.1016/j.jacadv.2026.103033)
Supplement: Supplemental Material [file mmc1.pdf]

SUPPLEMENTAL MATERIAL

Supplemental Figure 1. Covariate Balance Before and After Propensity-Score Matching. Standardized mean differences (SMD) of baseline clinical and echocardiographic covariates before (orange) and after matching (green). Dashed vertical lines denote SMD thresholds of 0.1 and 0.2.

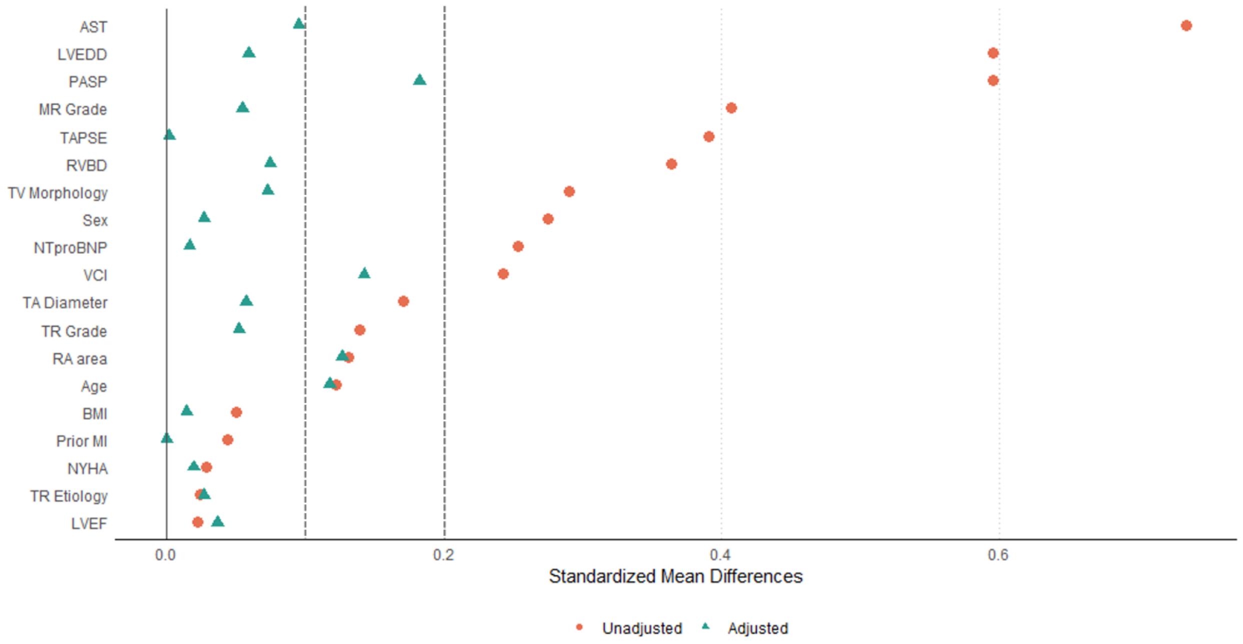

Supplemental Figure 2. Freedom From the Composite Endpoint After T-TEER and TTVA in the IPTW-Weighted Cohort. Survival curves depicting freedom from the composite endpoint of all-cause mortality or first HFH following T-TEER (blue) and TTVA (red). At 1 year, freedom from the composite endpoint was 75.2% (95% CI: 71.3–79.3) after T-TEER and 81.0% (95% CI: 71.9–91.3) after TTVA, with no significant difference between groups (IPTW-weighted Cox HR: 0.78; 95% CI: 0.42–1.46; *P*=0.440). Shaded areas represent 95% confidence intervals.

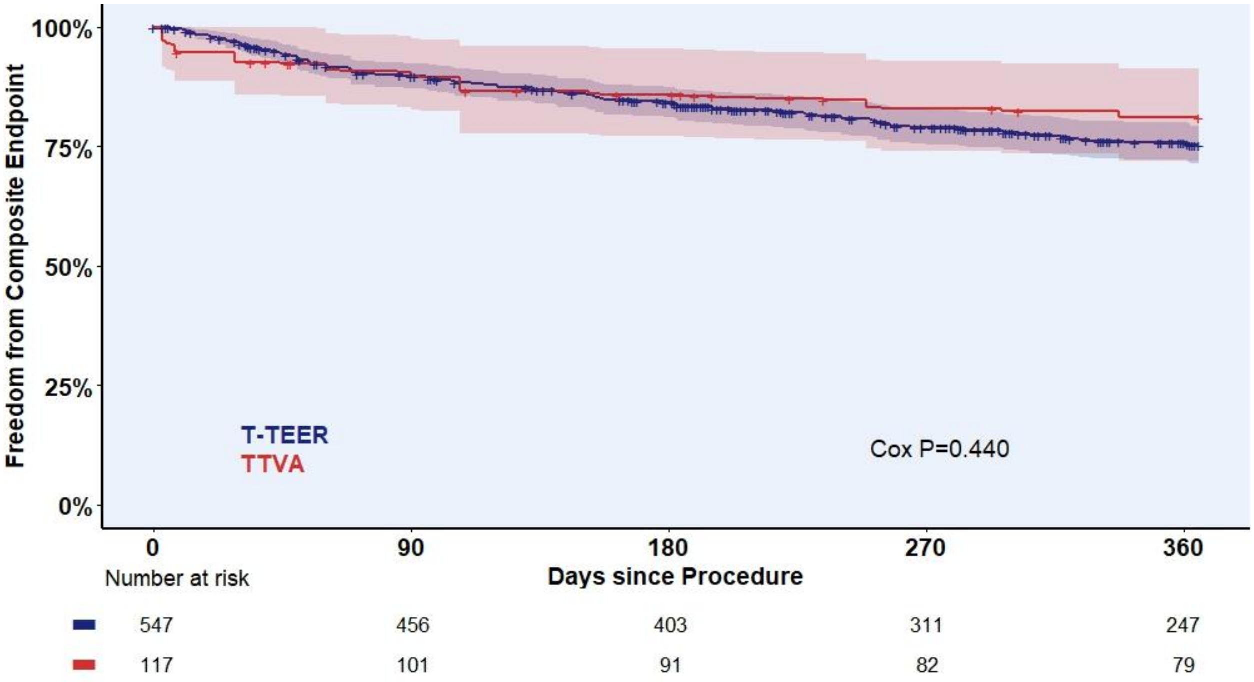

Supplemental Figure 3. Covariate Balance with Caliper 0.1 SD. Standardized mean differences (SMD) of baseline clinical and echocardiographic covariates before (orange) and after matching (green) using a stricter caliper specification (0.1 SD, yielding 104 patient pairs vs. 111 pairs in primary analysis). Dashed vertical lines denote SMD thresholds of 0.1 and 0.2.

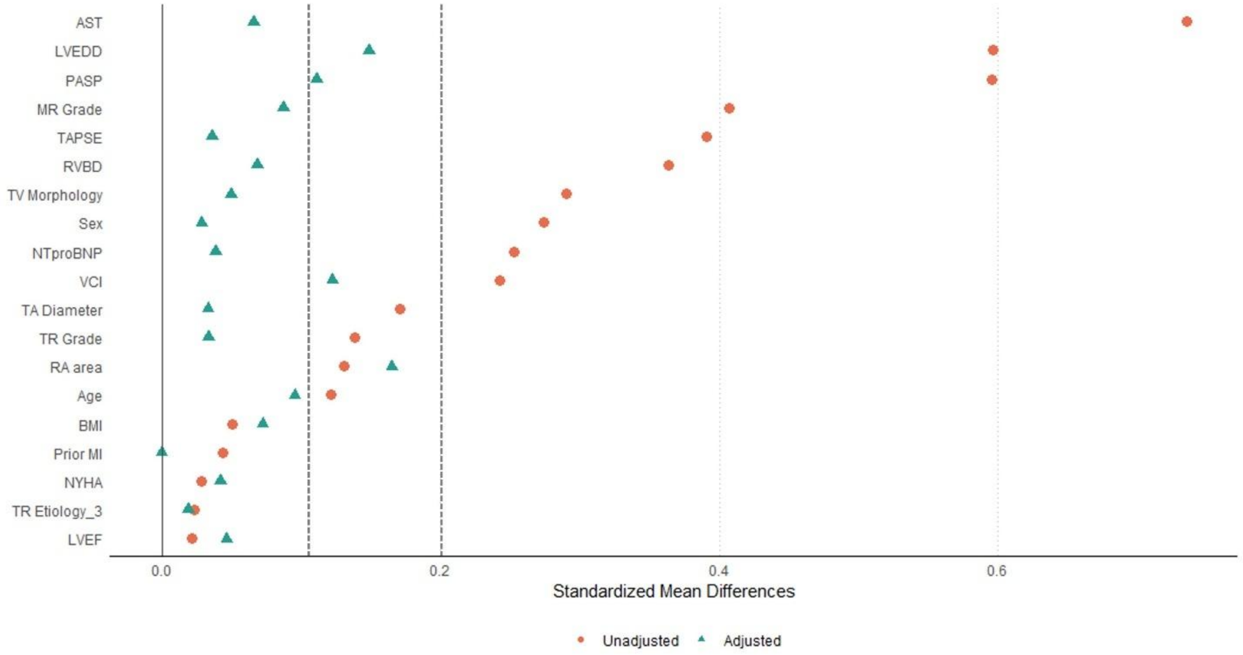

Supplemental Figure 4. Freedom From the Composite Endpoint After T-TEER and TTVA In the Matched Cohort With Caliper 0.1 SD. Survival curves depicting freedom from the composite endpoint of all-cause mortality or first HFH following T-TEER (blue) and TTVA (red). At 1 year, freedom from the composite endpoint was 76.0% (95% CI: 67.8–85.1) after T-TEER and 80.5% (95% CI: 73.0–88.8) after TTVA. Shaded areas represent 95% confidence intervals.

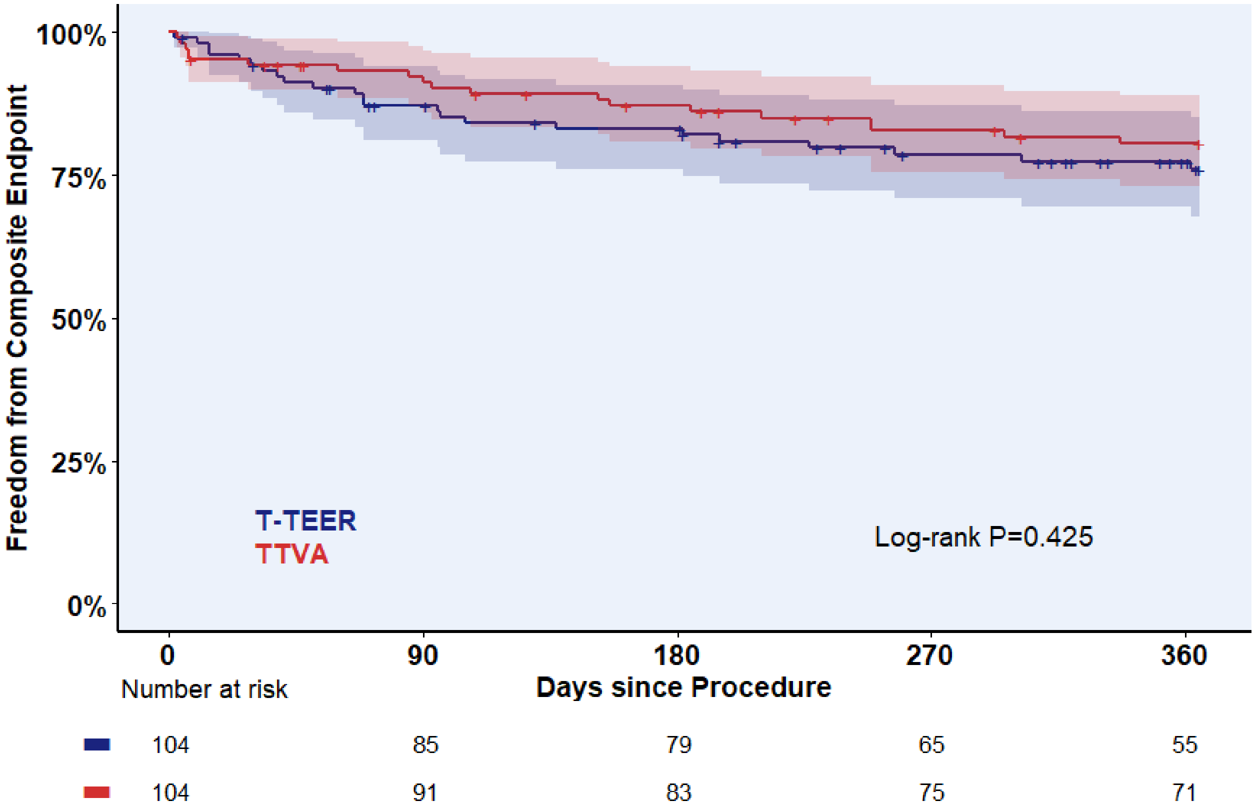

Supplemental Table 1. Baseline Matched Characteristics of T-TEER and TTVA

	**T-TEER N=111**	**TTVA N=111**	**P-Value**	**SMD**
Age, y	80 (75-83) (N=111)	80 (77-83) (N=111)	0.935	0.099
Female, %	90/111 (81.1)	87/111 (78.4)	0.738	0.000
BMI, kg/m^2	24.5 (21.3-28.3) (N=111)	25.5 (22.0-28.3) (N=111)	0.394	0.013
TRI-SCORE	4 (3-5) (N=111)	4 (3-6) (N=111)	0.334	0.141
EuroScore II, %	5.0 (2.7-8.4) (N=108)	4.1 (2.7-7.3) (N=111)	0.294	0.233
NYHA Class			0.702	0.000
I	0/0 (0.0)	0/0 (0.0)		
II	16/111 (14.4)	14/111 (12.6)		
III	83/111 (74.8)	88/111 (79.3)		
IV	12/111 (10.8)	9/111 (8.1)		
Prior HFH	70/110 (63.6)	68/111 (61.3)	0.821	0.000
**Comorbidities**				
Atrial fibrillation/flutter	101/111 (91)	103/111 (92.8)	0.806	0.000
Coronary artery disease	32/111 (28.8)	38/111 (34.2)	0.470	0.000
RV lead	22/111 (19.8)	28/111 (25.2)	0.422	0.000
Prior myocardial infarction	7/111 (6.3)	7/111 (6.3)	1.000	0.000
History of cardiac surgery	21/111 (18.9)	17/111 (15.3)	0.593	0.000
COPD	17/111 (15.3)	16/111 (14.4)	1.000	0.000
**Laboratory data**				
NT-proBNP, pg/mL	2383.0 (1470.0-4429.) (N=111)	2418.0 (1427.5-4233.5) (N=111)	0.473	0.017
AST, U/l	28 (23-35) (N=111)	29 (25-35) (N=111)	0.353	0.076
eGFR, mL/min/1.73 m²	43 (34-59) (N=111)	44 (34-58) (N=111)	0.986	0.010
eGFR <60 mL/min	85/111 (76.6)	84/111 (75.7)	1.000	0.000
**Medication**				
Loop diuretics	104/111 (93.7)	101/111 (91)	0.614	0.000
Furosemide-equivalent, mg	60 (35-95) (N=103)	40 (20-100) (N=111)	0.227	0.022
**Hemodynamic data**				
PASPinv	42 (35-49) (N=80)	42 (36-51) (N=95)	0.660	0.054

Loop diuretic doses were expressed as furosemide-equivalents (mg) using standard conversion factors (torsemide × 4). Values are n (%), median [Q1-Q3], or mean ± SD. AST = aspartate aminotransferase; BMI = body mass index; COPD = chronic obstructive pulmonary disease; eGFR = estimated glomerular filtration rate; EuroSCORE II =

European System for Cardiac Operative Risk Evaluation II; HFH: heart failure hospitalization; NT-proBNP = N-terminal pro–B-type natriuretic peptide; PM= pacemaker; RV = right ventricular; NYHA = New York Heart Association.

Supplemental Table 2. Baseline Matched Echocardiographic Assessment of T-TEER and TTVA

	**T-TEER N=111**	**TTVA N=111**	**P-Value**	**SMD**
RAA, cm^2	34.2 (29.9-4) (N=111)	36.4 (30.8-43.2) (N=111)	0.220	0.121
RV base diameter, mm	48.0 (41.5-52.0) (N=111)	48.0 (43.0-52.5) (N=111)	0.548	0.064
TAPSE, mm	16 (14-18) (N=111)	16 (13-19) (N=111)	0.948	0.002
Estimated PASP, mmHg	39 (32-47) (N=111)	35 (30-45) (N=111)	0.076	0.178
Inferior vena cava, mm	24 (21-29) (N=111)	25 (21-30) (N=111)	0.279	0.134
**Tricuspid Valve**				
Coaptation gap, mm	5.3 (4.0-8.0) (N=107)	6.0 (4.0-10.0) (N=110)	0.590	0.153
Tenting height, mm	6.0 (4.3-8.2) (N=109)	6.2 (4.4-8.8) (N=111)	0.567	0.057
Annular dimension SL, mm	41 (37-47) (N=111)	43 (39-46) (N=111)	0.366	0.041
Morphology				
I	46/111 (41.4)	47/111 (42.3)	0.754	0.000
II	3/111 (2.7)	3/111 (2.7)		
IIIa	10/111 (9)	9/111 (8.1)		
IIIb	27/111 (24.3)	35/111 (31.5)		
IIIc	14/111 (12.6)	10/111 (9)		
IV	11/111 (9.9)	7/111 (6.3)		
Tricuspid Regurgitation				
Grade			0.073	0.121
0	0/0 (0.0)	0/0 (0.0)		
1+	0/0 (0.0)	0/0 (0.0)		
2+	0/0 (0.0)	0/0 (0.0)		
3+	44/111 (39.6)	49/111 (44.1)		
4+	41/111 (36.9)	26/111 (23.4)		
5+	26/111 (23.4)	36/111 (32.4)		
Vena contracta width, mm	12.0 (9.3-15.0) (N=110)	13.0 (9.5-16.5) (N=111)	0.244	0.119
EROA, cm^2	0.58 (0.43-0.78) (N=106)	0.61 (0.48-0.86) (N=111)	0.221	0.063

Regurgitant volume, mL	42 (35-58) (N=105)	44 (32-56) (N=111)	0.969	0.014
Etiology				
Primary	0/0 (0.0)	0/0 (0.0)		
Secondary	102/111 (91.9)	99/111 (89.2)	0.646	0.000
Mixed	9/111 (8.1)	12/111 (10.8)		
Secondary TR Etiology			0.568	0.000
A-STR	23/101 (22.8)	27/99 (27.3)		
V-STR	78/101 (77.2)	72/99 (72.7)		
**Left Ventricle**				
LVEF, %	55 (45-60) (N=111)	55 (49-59) (N=111)	0.824	0.033
LVEDD, mm	43 (40-49) (N=111)	43 (40-49) (N=111)	0.779	0.057
Mitral regurgitation grade			0.115	0.115
0	22/111 (19.8)	13/111 (11.7)		
1+	60/111 (54.1)	70/111 (63.1)		
2+	24/111 (21.6)	27/111 (24.3)		
3+	5/111 (4.5)	1/111 (0.9)		
4+	0/0 (0.0)	0/0 (0.0)		

Values are n (%), median [Q1-Q3], or mean ± SD. A-STR = atrial secondary tricuspid regurgitation; EROA = effective regurgitant orifice area; LVEF = left ventricular ejection fraction; LVEDD = left ventricular end diastolic diameter; PASP = pulmonary artery systolic pressure; RAA = right atrial area; TAPSE = tricuspid annular plane systolic excursion; TR = tricuspid regurgitation; TV = tricuspid valve; V-STR = ventricular secondary tricuspid regurgitation.

Supplemental Table 3. Procedural and Safety Outcomes in Matched T-TEER and TTVA

	T-TEER N=111	TTVA N=111	P-Value	SMD
Procedure time, min	90 (66-131) (N=107)	197 (167-240) (N=103)	<0.001	1.863
Intraprocedural success	99/111 (89.2)	71/111 (64)	<0.001	0.291
TR Grade			0.018	0.199
0	5/106 (4.7)	4/111 (3.6)		
1+	48/106 (45.3)	34/111 (30.6)		
2+	40/106 (37.7)	37/111 (33.3)		
3+	10/106 (9.4)	27/111 (24.3)		
4+	3/106 (2.8)	8/111 (7.2)		
5+	0/106 (0)	1/111 (0.9)		
Number of devices	2 (2-2)	†		
Device type (PASCAL)		†		
PASCAL P10	13/111 (11.7)			
PASCAL Ace	98/111 (88.3)			
Device Position		†		
AS only	66/106 (62.3)			
PS only	6/106 (5.7)			
AS and PS combined	34/106 (32.1)			
Concomitant M-TEER	5/111 (4.5)	0/111 (0)	0.070	0.136
Number of anchors	†	17 (16–17) N=94		
Device type (Cardioband)	†			
C		2/108 (1.8)		
D		12/108 (10.8)		
E		27/108 (24.3)		
F		67/108 (60.4)		
Degree of cinching	†	5.5 (5.5–5.5) N=103		
Patients with any safety event	5/111 (4.5)	30/111 (27.0)	<0.001	
Total number of safety events	6	39		
SLDA*	4/111 (3.6)	†		
Anchor-detachment‡	†	2/111 (1.8)		
Access-site related venous or arterial injury (TVARC type ≤3 bleeding)	1/111 (0.9)	3/111 (2.7)	0.622	0.008
Bleeding (TVARC type >3)	1/111 (0.9)	8/111 (7.2)	0.035	0.145
Stroke/TIA	0/111 (0)	0/111 (0.0)	>0.999	
Acute kidney injury	0/111 (0)	8/111 (7.2)	0.007	0.182
RCA perforation	†	13/111 (11.7)		
RCA PCI	†	4/111 (3.6)		
Acute myocardial infarction	0/111 (0)	1/111 (0.9)	1.000	0.000

† Variable observed only in one treatment cohort; between-group comparison not applicable. * SLDA was identified intraprocedurally. ‡Anchor-detachment was identified at discharge. Values are n (%). PCI = percutaneous coronary intervention; RCA = right coronary artery; SLDA = single-leaflet device attachment; TIA = transient ischemic attack; TR = tricuspid regurgitation; TVARC = Tricuspid Valve Academic Research Consortium.

Supplemental Table 4. Baseline Characteristics of Matched T-TEER Patients With and Without Follow-up Echocardiography

	**T-TEER with Follow-up N=69**	**T-TEER without Follow-up N=42**	**P-Value**	**SMD**
Age, y	80 [77–83]	79 [74–83]	0.359	0.176
Female, %	54/69 (78.3)	36/42 (85.7)	0.470	0.000
BMI, kg/m^2	23.4 [21.1–27.2]	26.5 [22.2–31.3]	0.014	0.516
TRI-SCORE	4 [3–5]	4 [3–5]	0.189	0.192
EuroScore II, %	4.5 [2.5–8.1]	5.6 [3.1–10.3]	0.212	0.343
NYHA Class			0.349	0.062
I	0/69 (0.0)	0/42 (0.0)		
II	10/69 (14.5)	6/42 (14.3)		
III	54/69 (78.3)	29/42 (69.0)		
IV	5/69 (7.2)	7/42 (16.7)		
Prior HFH	41/68 (60.3)	29/42 (69.0)	0.470	**0.000**
NT-proBNP, pg/mL	2,224 [1,464–4,647]	2,759 [1,523–4,122]	0.580	0.051
eGFR <60 mL/min	52/69 (75.4)	33/42 (78.6)	0.876	0.000
Loop diuretics	62/69 (89.9)	42/42 (100)	0.043	0.179
Furosemide-equivalent, mg	40 [20–80]	80 [40–155]	0.014	0.307
LVEF, %	55 [45–63]	53 [42–60]	0.189	0.209
TR grade				
3+	28/69 (40.6)	16/42 (38.1)		
4+	27/69 (39.1)	14/42 (33.3)		
5+	14/69 (20.3)	12/42 (28.6)		
RV base diameter, mm	48 [41–52]	47.5 [43–52]	0.853	0.023
TAPSE, mm	16.0 [14.0–18.0]	15.5 [14.0–17.8]	0.443	0.089
Tenting height	6.0 [4.8–8.1]	6.0 [4.0–8.3]	0.410	0.091
Coaptation gap, mm	5.6 [4.0–7.9]	5.0 [4.0–8.0]	0.775	0.057
Mitral regurgitation grade			0.788	0.000
0	12/69 (17.4)	10/42 (23.8)		
1+	38/69 (55.1)	22/42 (52.4)		
2+	15/69 (21.7)	9/42 (21.4)		
3+	4/69 (5.8)	1/42 (2.4)		
4+	0/69 (0.0)	0/42 (0.0)		

Values are n/N (%) or median [Q1–Q3]. BMI = body mass index; eGFR = estimated glomerular filtration rate; HFH = heart failure hospitalization; LVEF = left ventricular ejection fraction; NT-proBNP = N-terminal pro–B-type

natriuretic peptide; RV = right ventricular; SMD = standardized mean difference; TAPSE = tricuspid annular plane systolic excursion; T-TEER = tricuspid valve transcatheter edge-to-edge repair; TR = tricuspid regurgitation.

Supplemental Table 5: Timing and Type of Reintervention within 1 Year following T-TEER or TTVA.

	**Reintervention**
**T-TEER**	
**#1**	During index procedure 1 PASCAL ACE was implanted with TR 3+ at discharge. After 57 days, reintervention with T-TEER was performed due to persistent severe, symptomatic TR.
**#2**	During index procedure 2 PASCAL ACE devices were implanted with TR 3+ at discharge. The patient was hospitalized for heart failure after 33 days and underwent TV surgery during the same hospital admission due to persistent severe, symptomatic TR.
**TTVA**	
**#1**	Successful Cardioband implantation with TR 2+ at discharge. Implantation of 2 PASCAL Ace devices (first in anteroseptal and second in posteroseptal commissure) after 147 days due to recurrent severe, symptomatic TR with worsening of right heart failure symptoms and preceding HFH 90 days post-procedure.
**#2**	Successful Cardioband implantation with TR 2+ at discharge. Implantation of 2 PASCAL Ace devices in anteroseptal commissure after 268 days due to recurrent severe, symptomatic TR.
**#3**	Unsuccessful Cardioband implantation with TR 3+ at discharge. Implantation of 2 PASCAL Ace devices (first in anteroseptal and second in posteroseptal commissure) after 133 days due to recurrent right heart failure symptoms.
