# Supplementary material for: Transcatheter Tricuspid Valve Annuloplasty vs Edge-to-Edge Repair: A Propensity-Matched Multicenter Comparative Analysis
Source: JACC Adv. 2026 Jul 21;5(8):103033. doi: 10.1016/j.jacadv.2026.103033 (PMC13396854; doi:10.1016/j.jacadv.2026.103033)
Supplement: PASTE Investigator List [file mmc2.docx]

**†PUBMED INDEXED INVESTIGATOR LIST:**

Florian Schindhelm, MD^k^, Tom Cahill, MD^q^, Kornelia Löw, MD^c^, Philipp Schlegel, MD^m^, Norbert Frey, MD^m^, Dominik Felbel, MD^j^, Stephanie Andreß, MD^j^, Amir Abbas Mahabadi, MD^k^, Volker Rudolph, MD^f^, Leonie Ziegler, MD^t^, Cornelia Deutsch, MD^u^, Violetta Hachaturyan, MD^u^, Peter Bramlage, MD^u^, Isabela Kast, MD^o^, Sebastian Ludwig, MD^h^, Roman Pfister, MD^a^, Stephan Baldus, MD^a^

^u^Institute for Pharmacology and Preventive Medicine, Cloppenburg, Germany
